# Reply to Bracke et al. Comment on “Prayag et al. Light Modulation of Human Clocks, Wake, and Sleep. *Clocks&Sleep* 2019, *1*, 193–208”

**DOI:** 10.3390/clockssleep3030026

**Published:** 2021-07-05

**Authors:** Abhishek S. Prayag, Mirjam Münch, Daniel Aeschbach, Sarah L. Chellappa, Claude Gronfier

**Affiliations:** 1 Lyon Neuroscience Research Center (CRNL), Waking Team, Inserm UMRS 1028, CNRS UMR 5292, Université Claude Bernard Lyon 1, Université de Lyon, 69000 Lyon, France; abhishek.prayag@inserm.fr; 2 Centre for Public Health Research, Massey University, Wellington 6140, New Zealand; M.Munch@massey.ac.nz; 3 Department of Sleep and Human Factors Research, Institute of Aerospace Medicine, German Aerospace Center (DLR), 51170 Cologne, Germany; Daniel.Aeschbach@dlr.de; 4 Faculty of Medicine, University of Bonn, 53127 Bonn, Germany; 5 Division of Sleep Medicine, Harvard Medical School, Boston, MA 02115, USA; schellappa@bwh.harvard.edu; 6 Medical Chronobiology Program, Division of Sleep and Circadian Disorders, Departments of Medicine and Neurology, Brigham and Women’s Hospital, Boston, MA 02115, USA

We thank Bracke and colleagues [1] for their commentary on our recent article ‘Light Modulation of Human Clocks, Wake, and Sleep’ by Prayag et al. (2019a) [1]. This gives us an opportunity to expand on our reported findings and interpretations and also to remind readers that mathematical models are aimed at simplifying complex biological mechanisms, and hence should be used parsimoniously and never be considered as absolute truths. We also underscore that results should always be interpreted cautiously, taking into account the experimental conditions the data were collected, and the non-visual responses investigated. We appreciate Bracke and colleagues’ [1] concern that our findings may be inappropriately interpreted by the non-specialist, in particular, Figures 4 and 5 of our review paper [2]. They raise two important points: (1) *“…*Figure 5 *could be misinterpreted by this audience as if monochromatic light sources need less melanopic-weighed irradiation than white light sources for the same response*”, and (2) *“…since the data for the monochromatic light sources are for pharmacologically dilated pupils, with normal pupil constriction (as for the white light irradiance response curves in Zeitzer et al., 2000) a much higher melanopic irradiance will be required for the same monochromatic response*”. In our review paper [2], Figure 4 is the illuminance-response curve for melatonin suppression (in response to 6.5 h polychromatic white light exposures) that we redrew from Zeitzer and colleagues (2000) to extend the illuminance axis. Figure 5 is the melanopic illuminance-response curve we modelled in one of our recent articles [3], derived from data obtained in response to 1.5 h of monochromatic light exposures [4]. In the interest of clarity, we would like to provide some elements of context and then address these aspects point- by-point. 

## 1. Elements of Context on the Importance of Experimental Conditions and on Mathematical Modelling

In their commentary, Bracke and colleagues [1] compare three melanopic irradiance-response curves that they derived from three studies where melatonin suppression was measured in response to either light at 460 nm (likely monochromatic), nine wavelengths (likely monochromatic), or fluorescent white light at 6500 K (see their Figure 1). Because the bibliographic references of the three studies were not reported, we cannot check whether these three studies were under the same experimental conditions (e.g., duration and timing of light exposure). Given that experimental findings are context-dependent, appropriate interpretation of the dataset and its limitations should be factored in when drawing conclusions. Thus, comparisons among datasets from different experimental conditions warrant further caution. It is for this very reason that readers of scientific articles, especially review articles, should always go back to the original papers in order to grasp the full picture and the limitations of each result. Indeed, the impact of the experimental conditions on the melanopic illuminance-response curve for melatonin suppression (and other non-visual responses) is discussed in our original paper [3]. We made it clear that the dataset [4] used to derive this melanopic-illuminance response curve had been obtained in conditions of (1) constant posture, (2) dilated pupils in order to keep the pupil size constant, (3) following a two-hour segment of dark adaptation, and (4) using a 90-min monochromatic light exposure starting around melatonin peak levels. Our finding that melanopic irradiance was the best predictor of melatonin suppression is in accordance with prior findings showing that monochromatic lights peaking between 460–480 nm induce the strongest non-visual responses in humans [4,5,6,7,8], in accordance with the melanopsin sensitivity peak at 480 nm [9]. The low EC10, EC50, and EC90 (0.2 and 2.5, 36.6 µW·cm^−^² melanopic irradiance) that we derived from our melanopic-illuminance curve is similar to that by Phillips et al. (2019) after two hours of light exposure following dim-light melatonin onset (0.3, 1.5, and 36.6 µW·cm^−^² melanopic irradiance, respectively). However, as we stressed in our original article, it is delicate to compare datasets collected using different protocols. It should be clear to the readers that the results displayed in Figures 4 and 5 of our review article have been obtained within specific laboratory settings, and that the associated conclusions are specifically, although not always exclusively, applicable to the experimental conditions under which data were collected. 

For the same reasons, it is fundamental to keep in mind that mathematical models, as the one we proposed in Figure 5 of our review [2,3] to express melatonin suppression as a function of melanopic illuminance, are rarely transposable to all conditions (experimental or real-life). However, such models are often applied to real-life conditions for the purpose of convenience but without accounting for their limitations. In our case, the mathematical equation linking light exposure to melatonin suppression will likely be different as a function of the duration of the light stimulus [10,11], intensity [12,13], timing [14,15,16,17], wavelength [4,5,8], and pattern of light exposure [18,19,20,21,22], but also as a function of prior light history [23,24,25,26]. These are also major predictors of non-visual response levels. 

Additional potential sources of variability that should also be taken into account before translating mathematical models into applications in real-life conditions include: age [8,27], chronotype [28,29], sex [30,31,32], individual differences in sensitivity to light [33,34,35], eye color [36] and lens density [8,37]. Individual sensitivity to non-visual light exposure may also depend on the time of the day and sleepiness [38,39]. The precise interactions of these factors and their impact on non-visual sensitivity to light are unknown, but they are likely non-linear and cannot be simply inferred [40]. For example, there are models of melatonin suppression as a function of light intensity [13] and duration of light exposure [10]. However, the melatonin suppression-level prediction of these models does not match the dynamics of suppression at a constant intensity (~9500 lux; Rahman et al., 2018), [21], or the dynamics of the suppression at constant duration (6.5 h Rahman et al., 2019) [22], most probably because of the non-linear interaction between intensity and duration. Altogether, applying models in conditions that deviate from those in which the underlying data were obtained, especially if those models have not been validated in other studies, is tempting but likely to convey inadequate predictions and conclusions.

## 2. Melatonin Suppression Depends on, and Is Predicted by, the Spectral Content in Melanopsin-Weighted Irradiance, Not on the Light Source *per se*

Bracke and colleagues [1] are of the view that polychromatic lights likely need less melanopic irradiance than monochromatic light sources to elicit melatonin suppression, and to that purpose, they show such a comparison between monochromatic and polychromatic light sources in Figure 1 of their commentary. However, Brown [41], compiling data from 18 studies in which light exposures were either from monochromatic or polychromatic light sources, recently showed that most non-visual responses in humans (including circadian phase shifting, increased alertness, and melatonin suppression) can be best-modeled by a similar four-parameter melanopic irradiance-response model (although with different parameters, as shown in Prayag et al., 2019b) [2]. Such findings are in accordance with our proposal that melanopic irradiance is the best predictor of melatonin suppression in humans [3]. Similar results have also been obtained in a study comparing multiple polychromatic lights in semi-naturalistic settings [42]. Therefore melatonin suppression, as well as other non-visual responses, depend on, and are best predicted by melanopsin-weighted irradiance, not by the type of light source *per se* (monochromatic, polychromatic, LEDs, fluorescent, etc.).

## 3. Influence of the Pupil Diameter on Melatonin Suppression

Bracke and colleagues raise an important point about the influence of the pupil diameter on melatonin suppression [1]. They argue that the melanopic irradiance-response model we propose [3] was obtained in conditions of a dilated pupil, and that “*a much higher melanopic irradiance will be required for the same monochromatic response*” in conditions of freely-moving pupils. This is a valid point as indeed a free pupil constricts in response to light exposure [43], and therefore, the amount of light falling on the retina is reduced proportionally to its diameter [44]. To address this point, we overlapped the melanopic sensitivity function for melatonin suppression we obtained [3] from data collected with dilated pupils [4] with the melanopic sensitivity functions we derived from the dataset by the same group, in the same experimental conditions, but obtained with freely-moving pupils [45]. We find an EC50 (irradiance inducing 50% of the maximal response) at 2.5 µW·cm^−^² melanopic irradiance with dilated pupils (Prayag et al., 2019c) [3], and at 9.6 µW·cm^−^² melanopic irradiance with freely-moving pupils. In other words, a ~4-fold higher melanopic irradiance is required in freely-moving pupil conditions, as compared to dilated conditions to elicit the same half-maximal response (melatonin suppression). This result is close to the proposal by Bracke and colleagues [1] of multiplying the illuminance in dilated conditions by 5× to 10× to account for normal pupil constriction. 

## 4. Conclusions

Altogether, we share the concerns by Bracke and colleagues [1] that fundamental human studies testing light responses can be misinterpreted and have to be approached with caution. We stress that mathematical modelling in biology is more useful to approach the underlying fundamental mechanisms than to predict responses to stimuli in other conditions. We advocate that the application of laboratory models to real-life conditions requires great caution given that the response levels are determined by a set of unique influences, and the impact of their combination is largely unknown, even in laboratory conditions. The only way we can efficiently bridge the gaps between fundamental research and applications, i.e., the laboratory world and the real world, is through qualitative exchanges and collaborations between biologists and engineers. We also support attempts to standardize light exposure protocols and description of light exposure data in publications see for example [46]. We are grateful to Bracke and colleagues for their thought-provoking commentary on our article, and we hope our reply will be useful and will foster collaborations between our disciplines. 

## References

[1] Bracke P., Van de Putte E., Ryckaert W.R. (2021). Comment Concerning the Effects of Light Intensity on Melatonin Suppression in the Review “Light Modulation of Human Clocks, Wake, and Sleep” by A. Prayag et al. Clocks&Sleep.

[2] Prayag A.S., Münch M., Aeschbach D., Chellappa S.L., Gronfier C. (2019). Light Modulation of Human Clocks, Wake, and Sleep. Clocks&Sleep.

[3] Prayag A.S., Najjar R.P., Gronfier C. (2019). Melatonin suppression is exquisitely sensitive to light and primarily driven by melanopsin in humans. J. Pineal Res..

[4] Brainard G.C., Hanifin J.P., Greeson J.M., Byrne B., Glickman G., Gerner E., Rollag M.D. (2001). Action spectrum for melatonin regulation in humans: Evidence for a novel circadian photoreceptor. J. Neurosci..

[5] Thapan K., Arendt J., Skene D.J. (2001). An action spectrum for melatonin suppression: Evidence for a novel non-rod, non-cone photoreceptor system in humans. J. Physiol..

[6] Cajochen C., Münch M., Kobialka S., Kräuchi K., Steiner R., Oelhafen P., Orgül S., Wirz-Justice A. (2005). High Sensitivity of Human Melatonin, Alertness, Thermoregulation, and Heart Rate to Short Wavelength Light. J. Clin. Endocrinol. Metab..

[7] Rüger M., St Hilaire M.A., Brainard G.C., Khalsa S.-B.S., Kronauer R.E., Czeisler C.A., Lockley S.W. (2013). Human phase response curve to a single 6.5 h pulse of short-wavelength light: Blue light phase response curve. J. Physiol..

[8] Najjar R.P., Chiquet C., Teikari P., Cornut P.-L., Claustrat B., Denis P., Cooper H.M., Gronfier C. (2014). Aging of Non-Visual Spectral Sensitivity to Light in Humans: Compensatory Mechanisms?. PLoS ONE.

[9] Berson D.M., Dunn F.A., Takao M. (2002). Phototransduction by retinal ganglion cells that set the circadian clock. Science.

[10] Chang A.-M., Santhi N., St Hilaire M., Gronfier C., Bradstreet D.S., Duffy J.F., Lockley S.W., Kronauer R.E., Czeisler C.A. (2012). Human responses to bright light of different durations: Light DRC in humans. J. Physiol..

[11] Prayag A.S., Jost S., Avouac P., Dumortier D., Gronfier C. (2019). Dynamics of Non-visual Responses in Humans: As Fast as Lightning?. Front. Neurosci..

[12] Cajochen C., Zeitzer J.M., Czeisler C.A., Dijk D.-J. (2000). Dose-response relationship for light intensity and ocular and electroencephalographic correlates of human alertness. Behav. Brain Res..

[13] Zeitzer J.M., Dijk D.-J., Kronauer R.E., Brown E.N., Czeisler C.A. (2000). Sensitivity of the human circadian pacemaker to nocturnal light: Melatonin phase resetting and suppression. J. Physiol..

[14] Khalsa S.B.S., Jewett M.E., Cajochen C., Czeisler C.A. (2003). A Phase Response Curve to Single Bright Light Pulses in Human Subjects. J. Physiol..

[15] St Hilaire M.A., Gooley J.J., Khalsa S.B.S., Kronauer R.E., Czeisler C.A., Lockley S.W. (2012). Human phase response curve to a 1 h pulse of bright white light: PRC to 1 h light pulses in humans. J. Physiol..

[16] Gabel V., Maire M., Reichert C.F., Chellappa S.L., Schmidt C., Hommes V., Viola A.U., Cajochen C. (2013). Effects of Artificial Dawn and Morning Blue Light on Daytime Cognitive Performance, Well-being, Cortisol and Melatonin Levels. Chronobiol. Int..

[17] Münch M., Nowozin C., Regente J., Bes F., De Zeeuw J., Hädel S., Wahnschaffe A., Kunz D. (2016). Blue-Enriched Morning Light as a Countermeasure to Light at the Wrong Time: Effects on Cognition, Sleepiness, Sleep, and Circadian Phase. Neuropsychobiology.

[18] Rimmer D.W., Boivin D.B., Shanahan T.L., Kronauer R.E., Duffy J.F., Czeisler C.A. (2000). Dynamic resetting of the human circadian pacemaker by intermittent bright light. Am. J. Physiol. Regul. Integr. Comp. Physiol..

[19] Gronfier C., Wright K.P., Kronauer R.E., Jewett M.E., Czeisler C.A. (2004). Efficacy of a single sequence of intermittent bright light pulses for delaying circadian phase in humans. AJP Endocrinol. Metab..

[20] Najjar R.P., Zeitzer J.M. (2016). Temporal integration of light flashes by the human circadian system. J. Clin. Investig..

[21] Rahman S.A., St Hilaire M.A., Gronfier C., Chang A.-M., Santhi N., Czeisler C.A., Klerman E.B., Lockley S.W. (2018). Functional decoupling of melatonin suppression and circadian phase resetting in humans: Decoupled melatonin suppression and phase resetting. J. Physiol..

[22] Rahman S.A., Wright K.P., Lockley S.W., Czeisler C.A., Gronfier C. (2019). Characterizing the temporal Dynamics of Melatonin and Cortisol Changes in Response to Nocturnal Light Exposure. Sci. Rep..

[23] Hébert M., Martin S.K., Lee C., Eastman C.I. (2002). The effects of prior light history on the suppression of melatonin by light in humans. J. Pineal. Res..

[24] Smith K.A., Schoen M.W., Czeisler C.A. (2004). Adaptation of Human Pineal Melatonin Suppression by Recent Photic History. J. Clin. Endocrinol. Metab..

[25] Chang A.-M., Scheer F.A.J.L., Czeisler C.A. (2011). The human circadian system adapts to prior photic history. J. Physiol..

[26] Chellappa S.L., Ly J.Q.M., Meyer C., Balteau E., Degueldre C., Luxen A., Phillips C., Cooper H.M., Vandewalle G. (2014). Photic memory for executive brain responses. Proc. Natl. Acad. Sci. USA.

[27] Higuchi S., Nagafuchi Y., Lee S., Harada T. (2014). Influence of Light at Night on Melatonin Suppression in Children. J. Clin. Endocrinol. Metab..

[28] Lack L., Bailey M., Lovato N., Wright H. (2009). Chronotype differences in circadian rhythms of temperature, melatonin, and sleepiness as measured in a modified constant routine protocol. Nat. Sci. Sleep.

[29] Maierova L., Borisuit A., Scartezzini J.-L., Jaeggi S.M., Schmidt C., Münch M. (2016). Diurnal variations of hormonal secretion, alertness and cognition in extreme chronotypes under different lighting conditions. Sci. Rep..

[30] Cain S.W., Dennison C.F., Zeitzer J.M., Guzik A.M., Khalsa S.B.S., Santhi N., Schoen M.W., Czeisler C.A., Duffy J.F. (2010). Sex Differences in Phase Angle of Entrainment and Melatonin Amplitude in Humans. J. Biol. Rhythm..

[31] Duffy J.F., Cain S.W., Chang A.-M., Phillips A.J.K., Munch M.Y., Gronfier C., Wyatt J.K., Dijk D.-J., Wright K.P., Czeisler C.A. (2011). Sex difference in the near-24-hour intrinsic period of the human circadian timing system. Proc. Natl. Acad. Sci. USA.

[32] Chellappa S.L., Steiner R., Oelhafen P., Cajochen C. (2017). Sex differences in light sensitivity impact on brightness perception, vigilant attention and sleep in humans. Sci. Rep..

[33] Phillips A.J.K., Vidafar P., Burns A.C., McGlashan E.M., Anderson C., Rajaratnam S.M.W., Lockley S.W., Cain S.W. (2019). High sensitivity and interindividual variability in the response of the human circadian system to evening light. Proc. Natl. Acad. Sci. USA.

[34] Cain S.W., McGlashan E.M., Vidafar P., Mustafovska J., Curran S.P.N., Wang X., Mohamed A., Kalavally V., Phillips A.J.K. (2020). Evening home lighting adversely impacts the circadian system and sleep. Sci. Rep..

[35] Chellappa S.L. (2021). Individual differences in light sensitivity affect sleep and circadian rhythms. Sleep.

[36] Higuchi S., Motohashi Y., Ishibashi K., Maeda T. (2007). Influence of eye colors of Caucasians and Asians on suppression of melatonin secretion by light. Am. J. Physiol. Regul. Integr. Comp. Physiol..

[37] Najjar R.P., Teikari P., Cornut P.-L., Knoblauch K., Cooper H.M., Gronfier C. (2016). Heterochromatic Flicker Photometry for Objective Lens Density Quantification. Investig. Opthalmology Vis. Sci..

[38] Münch M., Léon L., Crippa S.V., Kawasaki A. (2012). Circadian and Wake-Dependent Effects on the Pupil Light Reflex in Response to Narrow-Bandwidth Light Pulses. Investig. Opthalmology Vis. Sci..

[39] Daguet I., Bouhassira D., Gronfier C. (2019). Baseline Pupil Diameter Is Not a Reliable Biomarker of Subjective Sleepiness. Front. Neurol..

[40] Tekieh T., Lockley S.W., Robinson P.A., McCloskey S., Zobaer M.S., Postnova S. (2020). Modeling melanopsin-mediated effects of light on circadian phase, melatonin suppression, and subjective sleepiness. J. Pineal. Res..

[41] Brown T.M. (2020). Melanopic illuminance defines the magnitude of human circadian light responses under a wide range of conditions. J. Pineal. Res..

[42] Nowozin C., Wahnschaffe A., Rodenbeck A., de Zeeuw J., Hädel S., Kozakov R., Schöpp H., Münch M., Kunz D. (2017). Applying Melanopic Lux to Measure Biological Light Effects on Melatonin Suppression and Subjective Sleepiness. Curr. Alzheimer Res..

[43] Gamlin P.D.R., McDougal D.H., Pokorny J., Smith V.C., Yau K.-W., Dacey D.M. (2007). Human and macaque pupil responses driven by melanopsin-containing retinal ganglion cells. Vis. Res..

[44] Pokorny J., Smith V.C., Cavonius C.R. (1997). How much light reaches the retina?. Colour Vision Deficiencies XIII..

[45] West K.E., Jablonski M.R., Warfield B., Cecil K.S., James M., Ayers M.A., Maida J., Bowen C., Sliney D.H., Rollag M.D. (2011). Blue light from light-emitting diodes elicits a dose-dependent suppression of melatonin in humans. J. Appl. Physiol..

[46] Spitschan M., Stefani O., Gronfier C., Lockley S.W., Lucas R.J. (2019). Measuring light in chronobiology and sleep research. Clocks Sleep.

